# One Biosecurity is essential to implement One Health

**DOI:** 10.1093/biosci/biaf110

**Published:** 2025-07-23

**Authors:** Philip E Hulme, Michele S Dechoum, Franz Essl, Angeliki F Martinou, Aníbal Pauchard, Petr Pyšek, Helen E Roy, Andy W Sheppard, Montserrat Vilà, John R U Wilson

**Affiliations:** Centre for One Biosecurity Research, Analysis, and Synthesis, Lincoln University, Christchurch, Canterbury, New Zealand; Department of Pest-Management and Conservation, Lincoln University, Christchurch, Canterbury, New Zealand; Departamento de Ecologia e Zoologia, Programa de Pós-graduação em Ecologia of the Universidade Federal de Santa Catarina, Santa Catarina, Brazil; Division of BioInvasions, Global Change, and Macroecology, Department of Botany and Biodiversity Research, University of Vienna, Vienna, Austria; Laboratory of Vector Ecology and Applied Entomology, Joint Services Health Unit Cyprus, Akrotiri; Enalia Physis Environmental Research Centre, Nicosia; Climate and Atmosphere Research Centre, Cyprus Institute, Aglantzia, Nicosia, Cyprus; Laboratorio de Invasiones Biológicas, Facultad de Ciencias Forestales, Universidad de Concepción, Concepción, and with the Institute of Ecology and Biodiversity, Santiago, Chile; Czech Academy of Sciences, Institute of Botany, Department of Invasion Ecology, Průhonice; Department of Ecology in the Faculty of Science, Charles University, Prague, Czech Republic; UK Centre for Ecology and Hydrology, Wallingford; Centre for Ecology and Conservation, University of Exeter, Penryn, England, United Kingdom; CSIRO, Black Mountain Laboratories, Canberra, Australia; Estación Biológica de Doñana, Sevilla, Spain; Departamento de Biología Vegetal y Ecología, Universidad de Sevilla, Sevilla, Spain; South African National Biodiversity Institute, Kirstenbosch Research Centre, Cape Town; Centre for Invasion Biology, Department of Botany and Zoology, Stellenbosch University, Stellenbosch, South Africa

**Keywords:** epidemiology, food security, globalization, pandemics, quarantine

## Abstract

Although One Health and biosecurity both aim to protect the health of people, animals, and ecosystems from biological hazards, the two fields remain heavily siloed across distinct policy and research domains. One Health has yet to fully integrate environmental perspectives, especially biological invasions, into its workplan, whereas biosecurity lacks an effective inclusion of the social and health sciences, further hindering collaboration. One Biosecurity offers a vital interdisciplinary framework that bridges human, animal, plant, and ecosystem health sectors, fostering a stronger connection between biosecurity and One Health. This comprehensive approach spans the entire biosecurity continuum, from pre-border intelligence scans to border inspections and post-border incursion management, enabling more effective responses to the threats posed by biological invasions. By unifying these efforts, One Biosecurity will engage a broader group of multilateral organizations, bring together diverse stakeholders, and implement balanced strategies that better safeguard human health, agriculture production systems, and the natural environment.

The One Health concept initially arose at the nexus of wildlife, domestic animal, and human health around the emergence and management of zoonoses and called for a more inclusive approach to bring veterinary and human health closer together (Kahler 2004, Evans and Leighton 2014). Subsequently, the concept has been expanded and described in various ways (Prata et al. 2022a), with a global consensus definition only reached recently (Adisasmito et al. 2022). As was agreed by the Food and Agriculture Organization of the United Nations (FAO), the World Organization for Animal Health (WOAH), the United Nations Environment Programme (UNEP), and the World Health Organization (WHO), One Health is defined as “an integrated, unifying approach that aims to sustainably balance and optimize the health of people, animals, and ecosystems” (Adisasmito et al. 2022).

The term *biosecurity* first emerged as an approach to protect the agricultural sector from the threat of pests and pathogens but has also been the subject of multiple definitions (Hulme 2024b). However, one meaning agreed on by both WHO and FAO is “a strategic and integrated approach to analyzing and managing relevant risks to human, animal, and plant life and health and associated risks for the environment” (INFOSAN 2010). These risks include alien plant pests, animal pests and pathogens, pathogens capable of jumping from animals to humans (zoonoses), the release of genetically modified organisms and their products, and the management of invasive alien species and genotypes (Hulme 2020). The definitions of One Health and biosecurity are remarkably similar and imply considerable potential for complementarity. However, regardless of the similarity in definitions and the logic of an integrated approach, the research supporting either One Health or biosecurity has largely developed independently with remarkably little cross-fertilization over the last two decades (box 1). Biosecurity is scarcely mentioned in the One Health Joint Plan of Action (2022–2026) proposed by FAO, UNEP, WHO, and WOAH, and the only references are associated strictly with zoonoses and laboratory biosafety (FAO et al. 2022).

Box 1. **The disconnect between One Health and biosecurity**

Biosecurity relates to the research, procedures, and policies that cover the exclusion, eradication or effective management of the risks posed by the introduction of alien plant pests, animal pests and diseases, animal diseases capable of transmission to humans (zoonoses), the release of genetically modified organisms and their products, and the management of invasive alien species and genotypes (Hulme 2020). As such, it encompasses issues central to One Health, including the importation of human pathogens into a country and the spread of their alien vectors and reservoir hosts. To assess the extent to which the topic areas of One Health and biosecurity overlapped, a bibliometric analysis was undertaken on 22 October 2023 examining all articles archived on Web of Knowledge and published between 1 January 2000 and 31 December 2023. The search terms used were the keywords *One Health, biosecurity* or both terms together (*“One Health”* and *“biosecurity”*) to capture the frequency with which these two keywords were addressed jointly. The search was restricted to author-defined keywords together with Keywords Plus because this ensured the most relevant articles were retrieved and is known to be an effective way to capture the knowledge structure of scientific fields as well as an article's content (Hulme 2024b). Although this search strategy is conservative, there is no a priori reason why it would disadvantage either One Health or biosecurity proportionally more than the other, and because the analysis is comparative, any biases should be similar for each keyword allowing for general insights. (See figure 1).

**Figure 1.**
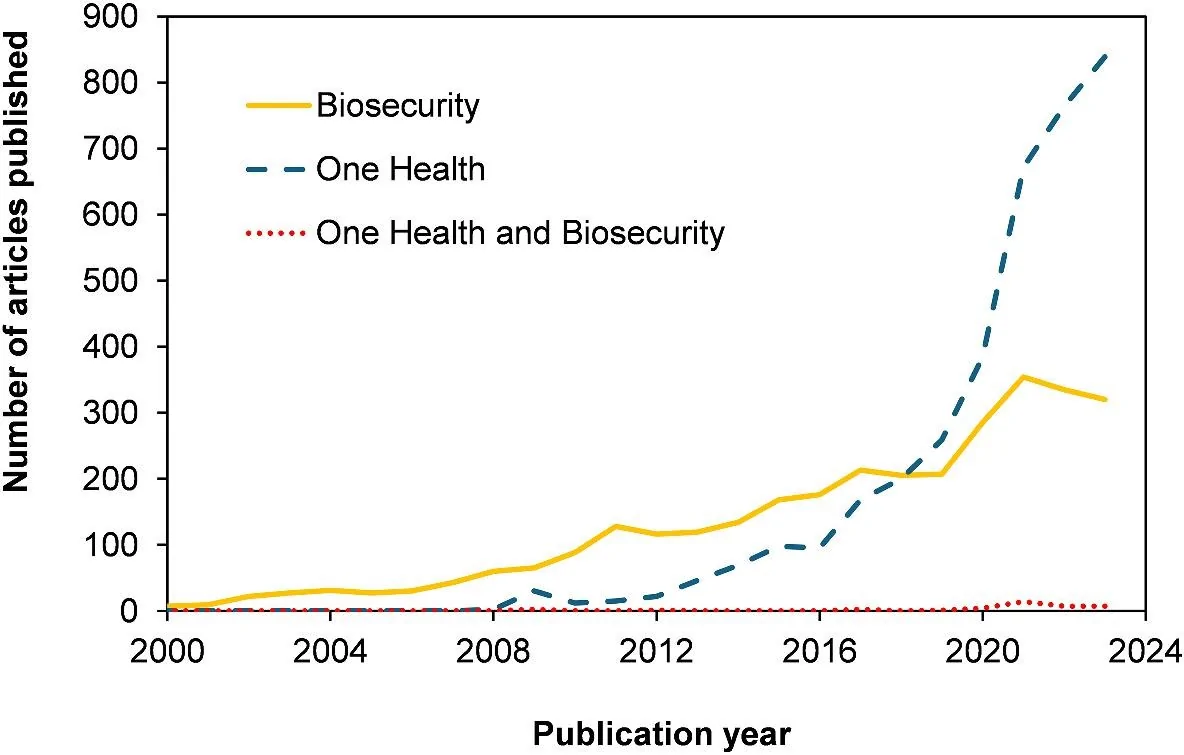
Despite an increasing number of articles addressing either One Health (3675) or biosecurity (3169) being published in the last two decades, few articles (38) examine the topics jointly.

Despite over 3000 articles being retrieved for either One Health or biosecurity less than 0.6% address the two subjects jointly and these were generally from a perspective of biosafety. Web of Science assigns each article to one or more of 252 Research Areas in science, social sciences, as well as the arts and humanities and is generally considered the best high-level classification scheme for detailed bibliometric analysis (Hulme 2024b). While 6 out of the top 10 research areas were common to One Health or biosecurity, One Health was focused more on human health whereas biosecurity focused more on the environment (*χ*^2^(13) = 1219.96, *p* < .001; see figure 2).

**Figure 2.**
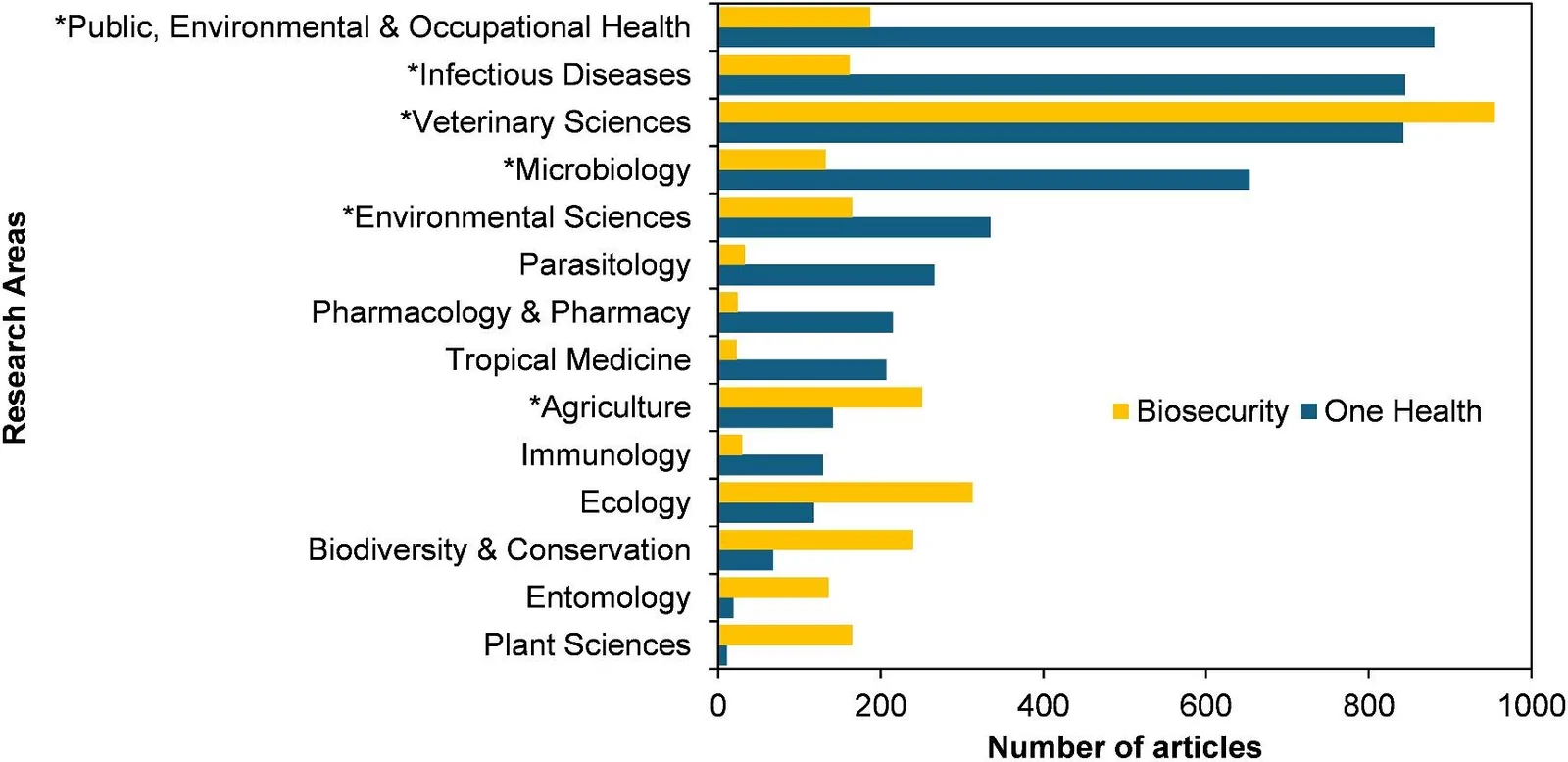
Notwithstanding the overlap in the top 10 research areas in the Web of Knowledge (with an asterisk), One Health focuses more on ‘Public, Environmental & Occupational Health’, ‘Infectious Diseases’, and ‘Microbiology’, whereas biosecurity focuses more on ‘Ecology, ‘Biodiversity & Conservation, and Plant Sciences.

The low frequency of articles jointly addressing One Health and biosecurity, together with marked differences in the primary research areas covered highlights a considerable disconnect between research on One Health and biosecurity.

The conceptual and operational similarities between biological invasions and the spread of human, animal, and zoonotic diseases (Hatcher et al. 2012, Ogden et al. 2019, Bertelsmeier and Oilier 2020, Hulme et al. 2020, Nuñez et al. 2020, Hulme 2021, Vilà et al. 2021) should allow much greater embedding of biosecurity into One Health and vice versa than has occurred to date. Despite similar definitions, there remain differences between One Health and biosecurity in the communities they service and in the dominant approaches applied to deliver their goals, the strategies for doing so, the sectors and taxa targeted, and the key players responsible for delivery (table 1). Why has there been such little cross-fertilization between these disciplines? There are at least three prime reasons for this disconnect. First, both disciplines exhibit strong siloing within different policy and research sectors. Second, the inadequate development or uptake of environmental perspectives in One Health limits the incorporation of threats from biological invasions. Third, the lack of interdisciplinarity in biosecurity has meant that social and health sciences perspectives are largely missing.

**Table 1.** Summary of some of the main characteristics of One Health and biosecurity with illustrative examples of key players and organizations, applications, strategies, and challenges.

**Characteristic**	**One Health**	**Biosecurity**
Definition	Integrated, unifying approach that aims to sustainably balance and optimize the health of people, animals, and ecosystems	Integrated approach to managing relevant risks to human, animal and plant life and health, and the environment
Scope	Broad and holistic, including human, animal, and environmental health	Focused on mitigating biohazards to human well-being, agriculture or biodiversity
Approach	Collaborative and interdisciplinary	Regulatory and preventive
Key Players	Epidemiologists, health advocates, public health workers, veterinarians	Aquaculturists, ecologists, farmers, foresters, land managers, veterinarians
Major focus	Disease prevention, health promotion, environmental stewardship	Containment and control of invasive alien species and pathway management
Example application	Public health, veterinary care, environmental management	Border controls, farm hygiene, prohibited species lists
Key goals	Promote health equity, sustainability, and biodiversity conservation	Protect economy, agriculture, human health and environment from biological invasions
Example strategies	Health education, prophylaxis, surveillance, vaccination	Biological control, inspection, quarantine, surveillance
Main challenges	Lack of implementation through policy and legislation	Balancing prevention with trade and travel
Intergovernmental organizations	FAO, UNEP, WHO, WOAH	Cartagena Biosafety Protocol CBD, IMO, IPPC, IUCN, WOAH
Illustrative cases	Zoonotic diseases (e.g., Ebola, highly pathogenic avian influenza, MERS, SARS, Zika)	Invasive alien species (e.g., fall armyworm, raccoon, ragweed, zebra mussel)

*Note:* One Biosecurity acts to build on similarities between One Health and biosecurity while also bridging any differences by integrating human, animal, plant, and ecosystem health more effectively within biosecurity.

The implementation of One Health has largely been focused on frameworks to foster more effective collaboration among specialists in human health, animal health, and ecosystem or environmental health at international or national levels (Blackburn et al. 2016, de La Rocque et al. 2019, Comizzoli et al. 2021, Rizzo et al. 2021, Ogunseitan 2022, Traore et al. 2023). Accordingly, concern has been expressed that there is a disconnect between the high-level promotion of One Health by multilateral organizations and governments and the reality on the ground in terms of delivering tangible solutions in health prevention and uniting health sectors (Enserink 2010, Ghai and Hemachudha 2022, Lefrançois et al. 2023). In contrast, the delivery of biosecurity outcomes by governments and industry is enshrined in international law, particularly through the Agreement on the Application of Sanitary and Phytosanitary Measures of the World Trade Organization, the International Maritime Organization's (IMO) International Convention for the Control and Management of Ships’ Ballast Water and Sediments, the FAO/WHO Codex Alimentarius, the International Plant Protection Convention (IPPC), and the FAO Code of Conduct for Responsible Fisheries (Hulme 2014a, 2021).

One possible reason for the difficulties in reaching effective implementation on the ground is that One Health remains dominated by issues pertaining to human and livestock health (particularly zoonoses), rather than the environment (Elnaiem et al. 2023, Zinsstag et al. 2023, Brown et al. 2024). In fact, a growing body of evidence highlights that the environment remains a poorly developed component of One Health, with many national and regional initiatives effectively ignoring it (Essack 2018, Khan et al. 2018, Humboldt-Dachroeden et al. 2020, Redford et al. 2022). One environmental issue that has been undervalued in One Health but should be a priority is biosecurity and the management of biological invasions (Chinchio et al. 2020, Hulme 2021). In addition to supporting global trade, biosecurity is acknowledged to be a central component in the delivery of current global initiatives to sustainably manage biodiversity and ecosystem services proposed by the Convention on Biological Diversity (CBD) through the Kunming–Montreal Global Biodiversity Framework (Milner-Gulland et al. 2021, CBD and IUCN 2024, Faulkner et al. 2024, Fu et al. 2024). This division between One Health and biosecurity in relation to the importance of environmental perspectives is also reflected in the underpinning science (box 1). Not surprisingly, research on One Health has been focused more on areas associated with human health (e.g., Public, Environmental, & Occupational Health, Infectious Diseases, and Microbiology), whereas research addressing biosecurity has been more strongly focused on the environment (e.g., Ecology, Biodiversity and Conservation, and Plant Sciences). Furthermore, even those studies that consider the environment within a One Health context have been focused primarily on wildlife reservoirs, zoonoses, or antimicrobial resistance rather than on biodiversity or ecosystem services (Hulme 2021).

One Health and biosecurity both aim to protect and sustain life by addressing complex, interlinked health, agricultural, and environmental challenges through integrated and interdisciplinary approaches. Both require input from diverse disciplines, including biology, environmental science, public health, agriculture, data science, and social sciences, to develop effective strategies (table 1). Considerable effort has been invested in determining what this might look like for One Health (Zinsstag et al. 2021, Prata et al. 2022b). In contrast, for biosecurity, there has only been limited cross-fertilization among human, animal, plant, and ecosystem health because of strong sectorial identities associated with specific international standards, individual economic sectors, specific research communities, and unique stakeholder involvement (Hulme 2014a). Furthermore, within biosecurity, the science supporting human, animal, plant, and ecosystem health sectors largely draws from a distinct literature base, with perspectives on ecosystem and plant health being largely disconnected from those addressing human and animal health (Hulme 2024b). Although an understanding of human behavior, societal values, and public perceptions are important to both One Health and biosecurity, this remains relatively poorly developed in the latter, especially with regard to plant and ecosystem health (Hulme et al. 2023). The lack of integration and collaboration may be a consequence of perceptions, with biosecurity considered within the field of veterinary medicine as an essential means to prevent and manage livestock diseases, whereas, in the context of public health, it is often regarded as synonymous with biosafety and preventing bioterrorism (Hulme 2024b). A more inclusive definition of biosecurity will challenge this limited conceptualization of biosecurity as it relates to human health and may hold the key to more innovative routes to operationalize One Health.

The foregoing points to the significant complementarity between One Health and biosecurity, where the interdisciplinary and collaborative frameworks of the former can be integrated with the existing policy instruments and implementation pathways of the latter. A closer union between biosecurity and One Health would connect a wider group of multilateral organizations and conventions, bring together a broader set of key players and stakeholders, and extend the breadth of strategies and approaches to address future challenges more effectively (table 1). However, such a closer union requires at least two major challenges to be overcome. The first is to increase awareness among public health professionals and policymakers that alien microbes, fungi, algae, plants, and animals pose a significant risk to health and human well-being. The second is to develop an interdisciplinary biosecurity culture that adopts a holistic and cross-sectorial approach recognizing the interdependencies among impacts on human, animal, plant, and ecosystem health. One route to overcome these two challenges and forge a closer union between One Health and biosecurity is the concept of One Biosecurity.

One Biosecurity has been recently defined as “an interdisciplinary approach to biosecurity policy and research that builds on the interconnections between human, animal, plant, and ecosystem health to effectively prevent and mitigate the impacts of invasive alien species” (Hulme 2020). A more integrated approach has major benefits by streamlining policy and legislation across different sectors, encouraging more holistic cross-sectoral assessments of threats, catalyzing interdisciplinary research programs targeting common goals, and enabling more effective communication of biosecurity messages to stakeholders and the public. Although invasive alien species are only one aspect of the environment that affect human health and well-being, they are significant agents of environmental change and a leading cause of biodiversity loss and therefore the cause of great damage to nature's contributions to people (defined as all the benefits and detriments that people get from their relationships with the rest of the living world) and good quality of life (Pyšek et al. 2020, Roy et al. 2024). Furthermore, biological invasions encompass issues central to One Health including the introduction of human pathogens into a country and the spread of their alien vectors and reservoir hosts. Nevertheless, it is important to emphasize that One Biosecurity is not an alternative to One Health but proposes a holistic approach to the threats posed by biological invasions that adversely affect biodiversity and also ultimately affect human health, well-being, and livelihoods.

Although the current concept of One Biosecurity emerged relatively recently, it has gained increasing traction at intergovernmental, national and subnational levels as a holistic approach that could address some of the current deficiencies in the management of biological invasions. For example, the Intergovernmental Science-Policy Platform on Biodiversity and Ecosystem Services (IPBES) has highlighted One Biosecurity as a valuable collaborative, multisectoral, and transdisciplinary framework to prevent and control invasive alien species (IPBES 2023). Similarly, the CBD and the World Conservation Union (IUCN) have stated that it would be beneficial to take a One Biosecurity approach to the management of biological invasions where national authorities responsible for managing risks to the environment and biodiversity, agriculture, and human health are working together (CBD and IUCN 2024). The FAO has recommended that members’ capacities are strengthened through a more integrated One Biosecurity approach that coordinates and transcends sector specific approaches and interventions on the upstream prevention and the sound management of risks to food security (FAO 2024).

In its review of the national biosecurity system, the New Zealand government aims to implement a One Biosecurity approach by creating an integrated, connected, and efficient biosecurity system to deliver better coordination across government, communities, and business while using technology and data for more informed, timely, and risk-based decisions (MPI 2023). The *National Biosecurity Policy and Action Plan 2022–2026* of the Nigerian Ministry of the Environment proposes One Biosecurity as a means to develop partnerships among relevant stakeholders to minimize and manage high-priority biosecurity threats to the integrity and reputation of the nation's primary produce (Ikeazor 2022). Furthermore, reviews of the national biosecurity policies in Malaysia (Shafie and Osman 2024) and South Africa (Wilson and Kumschick 2024) have recommended the incorporation of a One Biosecurity approach. Even in the absence of national regulations, there is evidence of individual industries recommending the adoption of a One Biosecurity approach. One of the key recommendations proposed by Australian Pork Limited in its national biosecurity strategy was to deliver the cultural change required to drive a One Biosecurity approach to facilitate ownership among a diverse array of stakeholders, including all levels of government, including health, tourism, community services and regional development, critical industry sectors, indigenous communities, and community groups (Australian Pork Limited 2021).

There is increasing momentum for One Biosecurity to bring together biosecurity practices needed for human, animal, plant, and environmental health whether to improve the monitoring of threats to plant health (Soubeyrand et al. 2024), provide a comprehensive risk management framework in animal health (Chan et al. 2021), or present more consistent public-facing health messaging (Elnaiem et al. 2023). One Biosecurity therefore has the scope to bridge the human, animal, plant, and ecosystem health sectors to stimulate more concrete incorporation of wider environmental perspectives into One Health.

To initiate more focused discussions on the role One Biosecurity can play in supporting One Health, we address the two main challenges limiting a closer union between biosecurity and public health. First, we set out why a more sustained effort is needed to raise awareness of the public health risks posed by alien species in terms of their direct impacts through communicable (e.g., zoonoses) and noncommunicable (e.g., allergies) diseases, as well as their wider indirect impacts on human well-being and livelihoods through impacts on ecosystem services and biodiversity. Second, by examining the complex web of interactions that link invasive alien species to human, animal, plant, and ecosystem health we point out the advantages of having public health clearly embedded into biosecurity thinking and highlight common tools and data that can be better employed to serve human, animal, plant, and ecosystem health sectors. Finally, we use the biosecurity continuum as an example of how a One Biosecurity approach to forecasting risks offshore, managing introduction pathways, inspecting people and goods at the border, and surveillance and management beyond the border can facilitate the implementation of One Health.

## Biological invasions require greater consideration in One Heath

Over the last decade, an increasing body of work has highlighted the threat that invasive alien species pose to human health (Conn 2014, Mazza et al. 2014, Schindler et al. 2015, Mazza and Tricarico 2018, Chinchio et al. 2020, Roy et al. 2023). In general, the direct effects of invasive alien species on human health have received the greatest attention in the literature but in this section, we also emphasise the indirect effects (especially on food security) that merit a more considered appraisal within One Health.

### Direct impacts of biological invasions on human health

The direct threats of invasive alien species on human health can be summarized into four broad issues that illustrate the importance of considering biosecurity within One Health. Specifically, we briefly describe how a biosecurity lens can support public health actions addressing: 1) zoonotic pathogens that are themselves often invasive alien species spread by the movement of infected humans and animals and by various other pathways; 2) invasive alien species that are vectors for zoonotic pathogens and parasites in regions where no such vectors were previously present; 3) invasive alien species acting as new hosts for existing pathogens; and 4) alien species directly affecting human health (e.g., as sources of allergens and toxins, or being themselves venomous). Rather than document a suite of examples, the aim is to illustrate how the process of applying biosecurity principles to the management of these threats can improve public health outcomes.

#### Human zoonotic pathogens as invasive alien species

Despite calls to consider SARS-CoV-2 and other pandemic diseases as biological invasions because of the similar processes of human transport across the globe, establishment often from a small inoculum in a new region, and subsequent spread in that region (Bertelsmeier and Oilier 2020, Nuñez et al. 2020, Vilà et al. 2021), few advances have been made to bridge disease and invasion biology (Hulme et al. 2020). In general, there has been less emphasis on pathogens themselves as invasive alien species compared with nonmicrobial taxa (Roy et al. 2017). For example, the global listing of 100 of the worst invasive alien species in the world does not include any human pathogens (Lowe et al. 2000). Similarly, despite the comprehensive nature of the recent IPBES *Thematic Assessment Report on Invasive Alien Species and their Control*, the detailed treatment of zoonotic pathogens as biological invasions was out of scope (IPBES 2023). But an understanding of the international movement of infected people and animals together with knowledge of the likelihood of further transmission at their destination is a critical public health issue that relies on sound biosecurity principles. For example, measles was introduced to Oceania by European settlers and although eradicated from Australia in 2014 and New Zealand in 2017, the importation of the virus into New Zealand in 2019 on an infected international traveler resulted in an outbreak which spread to Australia and Samoa resulting in over 80 deaths (Durrheim et al. 2024). Similarly, the Middle East respiratory syndrome coronavirus (MERS‐CoV) was first identified in Saudi Arabia in 2012, but an international traveler introduced it into South Korea in 2015, resulting in human-to-human transmission and an outbreak that killed 89 people (Lee 2016). Public health measures therefore need to consider the biosecurity risks posed by imported cases and to better understand the origin, number, and frequency of potential introduction events that could initiate or further exacerbate a disease outbreak. Without such knowledge, public health measures may be ineffective. The exceptionally porous international borders in West Africa meant that, during the 2014 Ebola outbreak, as long as one country experienced intense transmission of the pathogen, the neighboring countries remained at risk, no matter how strong their own public health measures had been (WHO 2015). At a global scale, the goal to reduce hepatitis C infections worldwide by 90% by 2030 (Cooke et al. 2019) needs to recognize that a significant proportion of cases reported by countries are imported (ECDC 2024). Furthermore, mosquito-borne diseases such as Zika, dengue, and chikungunya are frequently imported into countries where these diseases are not endemic, which, with the presence of appropriate alien mosquito vectors, has led to short-term outbreaks (Silburn and Arndell 2024). Where outbreaks have not occurred, it is thanks to the absence of appropriate vectors. An effective public health response therefore relies on an understanding of biological invasions and the implementation of appropriate biosecurity.

Although the international movement of humans is a key driver of the establishment of invasive alien zoonotic pathogens into new regions, other pathways of introduction also exist. The international trade in animals is a well-recognized means through which human pathogens have been introduced worldwide. An outbreak of monkeypox in 81 humans in the United States in 2003 was traced back to a child being bitten by pet prairie dogs (*Cynomys ludovicianus*) that had contracted the virus from Gambian giant rats (*Cricetomys gambianus*) imported into the United States for the pet trade (Brown 2008). The prion disease responsible for bovine spongiform encephalopathy that can cause variant Creutzfeldt–Jakob disease in people following the consumption of meat products was introduced to the United States and Canada through the import of a single infected cow (Lewis et al. 2010).

However, trade in other products can also lead to the introduction of pathogens that subsequently infect humans. In the 1970s and 1980s, contaminated blood products imported into the United Kingdom from the United States resulted in the infection of 30,000 people with hepatitis C or HIV virus, resulting in several thousand deaths to date (Lancet Infectious Diseases^ 2024^). There are a wide range of emerging pathogens that can be transmitted through the transfusion of blood and associated products such that international blood imports are likely to pose a route for the introduction of nonendemic pathogens to low-income countries where routine testing is less frequent (Fong 2020). The import of commodities sourced from animals may also present a risk of introducing human pathogens. For example, anthrax outbreaks have been associated with the importation of wool and animal hides, and these commodities can pose an emerging threat to countries claiming to be free of the disease (Wattiau et al. 2008). Finally, there are cases where pathogens can be spread into new regions without any association with human or animal products. For over a century, South America was cholera free, but an epidemic began in 1991 as a result of toxigenic *Vibrio cholerae* O1 from Asia being introduced through contaminated ballast water into Peruvian ports by international vessels (Seas et al. 2000). National and international policies addressing biosecurity have increasingly applied frameworks to manage introduction pathways of invasive alien species (CBD and IUCN 2024), and there would seem to be a strong case for developing similar frameworks relating to human health.

#### Alien species as vectors of zoonotic pathogens and parasites

The archetypal invasive alien threats to public health are mosquitoes (e.g., *Aedes, Anopheles, Culex*), which have been inadvertently introduced into new regions where they can vector zoonotic diseases such as malaria, dengue, chikungunya, and West Nile virus (Cuthbert et al. 2023). The annual global public health cost of just two invasive mosquitoes (*Aedes aegypti* and *Aedes albopictus*) has been estimated to be almost $2 billion (Roiz et al. 2024). Although there are many other invasive hematophagous arthropods that are vectors of zoonotic diseases (e.g., phlebotomine sand flies, culicoides midges, body lice, fleas, hard and soft ticks), their impacts are less well understood (Hulme 2014b, Cuthbert et al. 2023). In 2010, the phlebotomine sand fly (*Lutzomyia longipalpis*), which is a vector for canine and human leishmaniasis, was recorded for the first time in Uruguay and led to an outbreak of canine visceral leishmaniasis that also constitutes an emerging zoonotic risk to the human population (Satragno et al. 2017). Following its introduction in 2017, the Asian longhorned tick (*Haemaphysalis longicornis*) has spread in the United States, where it has the potential to transmit endemic and emerging zoonotic pathogens when biting humans (Molaei et al. 2022). Cats were introduced into New Zealand by European settlers in the nineteenth century and several emerging zoonotic pathogens including *Bartonella henselae, Bartonella clarridgeiae*, and *Rickettsia felis* have been found in cat fleas (*Ctenocephalides felis*), suggesting humans are likely to be infected (Kelly et al. 2005). The pathway of introduction of these pathogens is unclear but the biosecurity procedures for cat imports focus on preventing the entry of external (ticks and fleas) and internal (cestodes and nematodes) parasites, as well as respiratory diseases rather than zoonotic pathogens (MPI 2021). Without standard serological assessment of imported animals, it is likely that pathogens will breach international borders when hosts are asymptomatic.

#### Alien species as hosts of zoonotic pathogens and parasites

By far the most frequent role that invasive alien species play in zoonoses is as either primary or secondary hosts of existing or emerging pathogens and parasites (Roy et al. 2023). Vertebrates represent the vast majority of alien zoonotic hosts, particularly mammals and waterfowl, although bats and primates, despite often being the focus of One Health, are poorly represented because of their infrequent invasions (Zhang et al. 2022). Alien vertebrates are important reservoir hosts of diseases vectored by arthropods (e.g., Lyme disease, tularaemia, rickettsial infections) but also through their feces and urine, they contaminate the environment with pathogens responsible for salmonellosis, toxoplasmosis, and leptospirosis (Hulme 2014b). Several mollusks, especially snails, are intermediate hosts for platyhelminths and nematodes that infect humans (Mazza et al. 2014). Alien zoonotic hosts are a particular cause for concern because they thrive in anthropogenic environments, increasing the risk of transmission to humans and potentially opening the door to the establishment of new emerging diseases with which they have coevolved in their own native ranges (Hulme 2014b). Furthermore, at least for mammals, there is an indication that alien populations can be more widely infected by zoonotic pathogens than sympatric native host populations (Roy et al. 2023). Alien species have increased the frequency of zoonosis events worldwide, with regions having a high number of alien species that are hosts to zoonotic disease-causing agents also unsurprisingly having many zoonosis events that are correlated with the timing of alien host introductions (Zhang et al. 2022). Through its strong focus on prevention, a One Biosecurity approach could help avert future pandemics, potentially resulting in annual savings of several billion dollars to the global economy (Bernstein et al. 2022).

Although they are not technically zoonotic, there is even evidence for alien plants acting as hosts of human pathogens. *Cryptococcus* fungi are a leading cause of cryptococcosis in humans and animals and for *Cryptococcus neoformans* var. *gattii*, exposure to its host *Eucalyptus camaldulensis*, an invasive alien tree in many parts of the world, is necessary for infection (Ellis and Pfeiffer 1990). Many North American pines are invasive in New Zealand and Australia and their bark appears to be a suitable host for *Legionella longbeachae* and, when used to produce compost and potting mix, has resulted in Legionnaires’ disease among gardeners in both countries (Chambers et al. 2020).

#### Nonzoonotic impacts of alien species on human health

Alien species can affect human and animal health directly through the production of allergenic pollen and dust, toxins, and venoms, as well as through physical injuries (Nentwig et al. 2017, Lazzaro et al. 2018). The significance of these impacts on human health are difficult to gauge because reports are often anecdotal and the scale of harm is hard to quantify. However, it is likely that their overall impact on human health is several orders of magnitude less than the impact of alien species that are vectors or reservoir hosts of zoonoses (Bradshaw et al. 2016), Furthermore, similar or even greater harm to human health may arise from sympatric native species (evidence that alien species are causing distinct impacts is therefore important). Nevertheless, there are several alien species that stand out in terms of their nondisease related public health risk. Following its arrival from South America in the twentieth century, the red imported fire ant (*Solenopsis invicta*) now infests most of the southern United States where between 30% to 60% of residents are stung painfully each year, in some cases resulting in potentially life-threatening manifestations of bronchospasm, laryngeal oedema, or hypotension (Kemp et al. 2000). Common ragweed (*Ambrosia artemisiifolia*) was introduced to Europe in the nineteenth century and has become widespread on the continent (Essl et al. 2015) where today some 13.5 million persons suffer from Ambrosia-induced allergies (allergic rhinitis and severe asthma) that are estimated to cost US$80 billion annually (Schaffner et al. 2020). The aptly named nomad jellyfish (*Rhopilema nomadica*) invaded the eastern Mediterranean from the Red Sea through the Suez Canal in 2011 and is now the main source of jellyfish stings in the region resulting in swelling, whiplike lesions and blisters with some permanent scarring (Edelist et al. 2023).

### Indirect impacts of biological invasions on human health

The ramifications of biodiversity loss on human health are as yet poorly known but mounting evidence points to a risk of increasing emergence of zoonotic diseases (Keesing and Ostfeld 2021). For this reason, the CBD launched its global action plan on biodiversity and health, which aims to mainstream biodiversity and health interconnections into national policies (CBD 2024). Invasive alien species are one of the major causes of biodiversity loss across all regions of Earth (IPBES 2023) and therefore may be expected to have indirect impacts on human health. The WHO defines health as a “state of complete physical, mental, and social well-being and not merely the absence of disease or infirmity” (Schramme 2023), and therefore, health is embedded in the quality of the material, nonmaterial, and regulating services to people. Biological invasions often degrade the quality of nature's contributions to people, whether these are material contributions such as food and water, regulating contributions such as pollination and pest control, or nonmaterial contributions such as recreation and sense of place (Vilà and Hulme 2017, IPBES 2023). The negative impacts biological invasions have on ecosystem services can certainly have downstream implications for human health, but they have been little explored in any quantitative manner.

The United Nations General Assembly adopted 17 Sustainable Development Goals (SDGs) that ultimately aim to improve human health and well-being through societal, economic, and environmental transformation (Weeks et al. 2023). Compared with One Health, research addressing biosecurity addresses a much broader range of SDGs (box 2). This is especially marked for those SDGs relating to wider environmental impacts on human well-being such as climate change (SDG13, climate action) and the loss of biodiversity on land (SDG15, life on land) and in water (SDG14, life below water) but also food security (SDG02, zero hunger), whereas One Health research is largely focused on the direct outcomes of improved public health (SDG03, good health and well-being). The dominant focus on disease and infirmity rather than physical, mental, and social well-being in One Health highlights a major gap in its broad aim to optimize the health of people, animals, and ecosystems. However, it is noticeable that biosecurity research has not advanced gender issues (SDG05) as much as One Health, despite evidence that invasive alien species can disproportionally affect women more than men (Christie et al. 2025).

Box 2. **Differential contribution of One Health and biosecurity research to the UN Sustainable Development Goals**

Categorizing the articles arising from a bibliometric search (described in box 1) using the Web of Science Sustainable Development Goal classes (Lenzner et al. 2024) reveals marked difference between One Health and biosecurity (*χ*^2^(15) = 1551.32, *p* < .001, see figure 3). Overall, articles in One Health have a stronger emphasis on good health and well-being (SDG03) and clean water and sanitation (SDG06), whereas biosecurity articles have a stronger representation in life on land (SDG15), climate action (SDG13), life below water (SDG14), zero hunger (SDG02), and sustainable cities and communities (SDG11).

**Figure 3.**
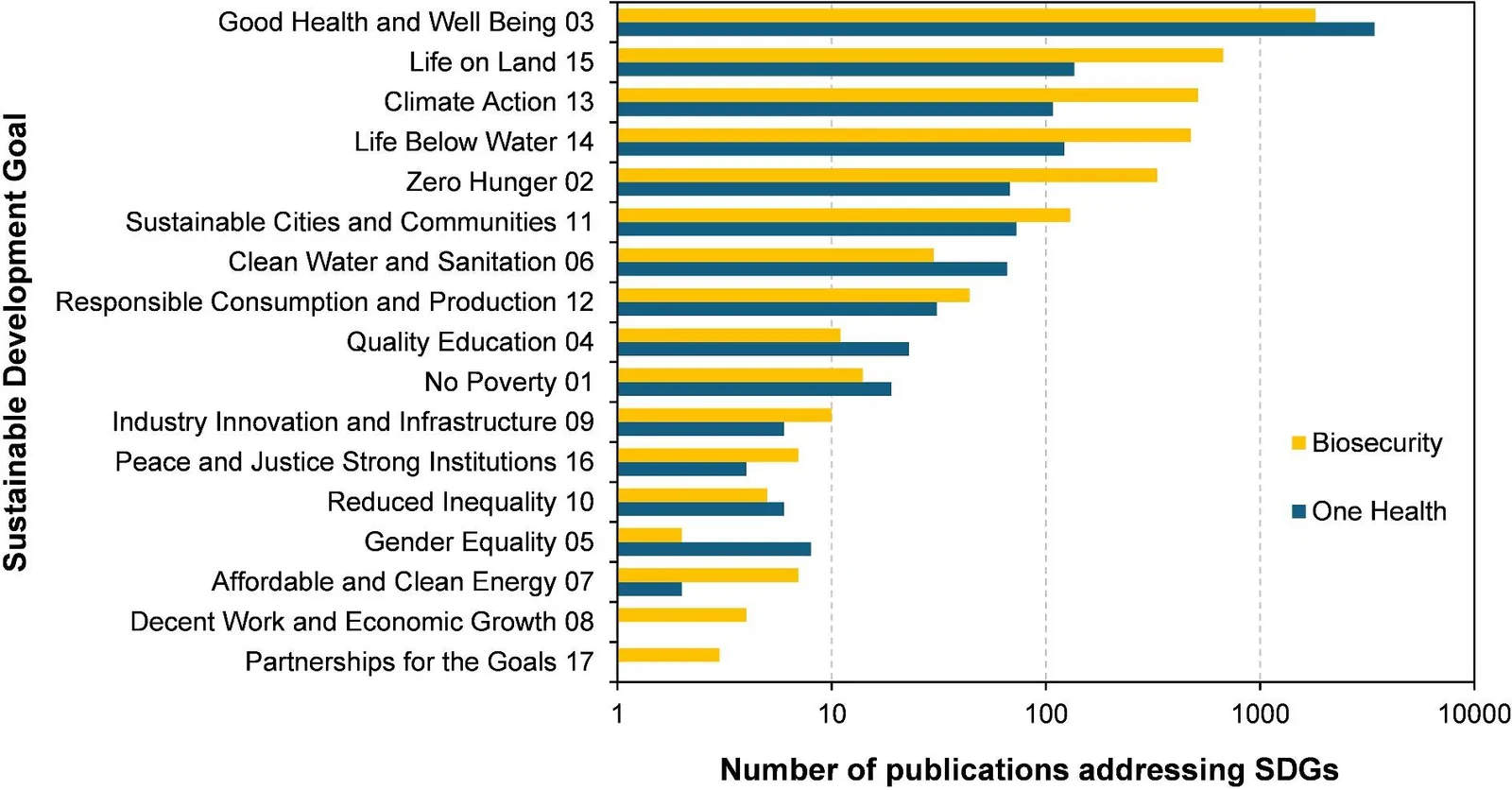
Different emphasis on the UN Sustainable Development Goals in research articles addressing One Health and biosecurity.

The material contribution that has received most attention in terms of the impact of biological invasions on human health is the provision of food for human consumption (IPBES 2023), which is integral to SDG02, zero hunger. Alien pathogens and parasites can have a major impact on food security through their effects on livestock health and include several major transboundary diseases such as foot and mouth disease, African swine fever, and highly pathogenic avian influenza (French 2017). Rinderpest, an infectious viral disease of cattle was likely introduced into Ethiopia by Italian troops in the late nineteenth century, and the subsequent plague wiped out livestock and other ungulates, contributing to perhaps 400,000 human deaths in the Great Ethiopian Famine of 1888–1892 (Pankhurst 1966). However, some of the most dramatic examples of alien pathogens resulting in famine stem from plant health. The Irish potato famine in the nineteenth century was the result of potato blight (*Phytophthora infestans*) being introduced from the United States, causing massive crop failure and an estimated death toll of 1.0–1.5 million people (Powderly 2019). The Bengal famine in 1943 was driven by production losses in rice of 40%–90% caused by a virulent strain of the brown spot fungus (*Cochliobolus miyabeanus*) and resulted in an estimated 2 million deaths (Padmanabhan 1973).

Outside of large-scale famines, the indirect consequences of alien crop pest, pathogens, and weeds on human health through malnutrition have not been quantified (IPBES 2023). Models of the impact of African swine fever, a virulent disease of pigs that recently spread into Eastern Asia and subsequently Europe, suggest declines in calorie availability in most regions affected (Mason-D'Croz et al. 2020). Alien pests and pathogens of agricultural crops are expanding their global ranges (Bebber et al. 2014) and challenge food security by reducing crop yields and quality (Fried et al. 2017). Emerging plant pathogens have been estimated to lead to production losses worth over US$1.4 billion annually on food crops across Africa (Sileshi and Gebeyehu 2021). These invasions exacerbate existing problems of poverty (SDG01, no poverty) that result in insufficient quantity or quality of food that has a major impact on human health (Friel and Ford 2015) with undernutrition an underlying cause in 45% of all child deaths worldwide (Mark et al. 2020). Alien species pose a particular problem in low-income economies where impacts are often on staple crops (e.g., cassava, sorghum) that are essential to the nutrition of the local community (Pratt et al. 2017). Even a single invasive alien pest can cause major impacts on food production and local livelihoods. The fall armyworm (*Spodoptera frugiperda*) is a destructive caterpillar native to tropical and subtropical regions of the Americas that is now established as an alien pest in more than 40 countries, with the estimated annual losses to maize yields being $13 billion (Mendesil et al. 2023). Local communities in Africa have been found to experience greater hunger following fall armyworm invasion (Tambo et al. 2021). Even where crops are not staples but commodities for export, the health of local communities can be affected where the management of alien pests, pathogens, and weeds require more intensive application of pesticides (Cocco 2016, Yang et al. 2021).

The consequences for public health of alien species affecting regulating and nonmaterial contributions of nature to people are less well understood, but two examples give a flavor of these effects (Vilà and Hulme 2017). Rather than regulating pest numbers, certain alien plants such as water hyacinth (*Pontederia crassipes*), Japanese barberry (*Berberis thunbergii*), multiflora rose (*Rosa multiflora*), and lantana (*Latana camara*) can increase pest problems (SDG03, good health and well-being) by providing habitats for insect vectors of human pathogens increasing their local density and the incidence of infection (Mazza et al. 2014, Hulme 2020). Urban trees provide an important cultural service in cities by combating air pollution (SDG11, sustainable cities and communities), but the loss of 100 million street trees because of emerald ash borer (*Agrilus planipennis*) invasion across 15 US states was associated with over 6000 additional human deaths related to cardiovascular and lower-respiratory-tract illness, with the effect being greater as the invasion progressed (Donovan et al. 2013). Similarly, a large cohort study was necessary to highlight that children who lived in areas with natural vegetation during most of their childhood experienced fewer asthma symptoms than those who lived most of their early life closer to large stands of invasive alien species such as gorse (*Ulex europeaus*) and Monterey pine (*Pinus radiata*) in New Zealand (Donovan et al. 2018). Greater effort is needed to quantify the human health impacts of biological invasions and this requires a more rigorous appraisal such as mortality, morbidity, years of potential life lost or disability-adjusted life years instead of qualitative scoring of the possible magnitude of impacts (Thacker et al. 2006). A major gap in the communication of the impacts of biological invasions on public health is the absence of quantitative studies assessing their long-term effects on the complete physical, mental, and social well-being of the public, as well as on the prevalence of disease and infirmity.

## Cross-sector impacts require biosecurity to be interdisciplinary

The foregoing has pointed to the multiple direct and indirect ways biological invasions can affect human health and well-being. Despite the important role that biosecurity plays across all the SDGs, approaches to assessing the risk and management of invasive alien species are often sector or discipline specific, as is illustrated by the WOAH Terrestrial and Aquatic Animal Health Codes (Thompson et al. 2024), the IUCN Environmental Impact Classification for Alien Taxa (Hawkins et al. 2015), and the IPPC Framework for Pest Risk Analysis (FAO 2019). Not every organism introduced to a new region will affect multiple sectors, nor will human health always be affected, but there are sufficient examples of alien species causing multisectoral impacts with consequences for the physical, mental, and social well-being of the public that such interactions need to be given serious consideration (table 2). Often, the impacts on human health parallel those on animal health because of shared parasites and pathogens or direct harm because of stings, bites, toxins, or allergens. In contrast, impacts on plant and ecosystem health can be quite different from those on animals and humans.

**Table 2.** Examples of invasive alien plants and animals known to have impacts across human, animal, plant, and ecosystem health.

**Organism**	**Name**	**Human health**	**Animal health**	**Plant health**	**Ecosystem health**
*Gymnodinium catenatum*	Naked dinoflagellate	Toxic microalgae that cause paralytic shellfish poisoning	Mass mortality at shrimp farms due to bloom toxins	Contaminates algal aquaculture production	Blooms intercept light and affect benthic species
*Datura stramonium*	Jimson weed	Humans poisoned by eating flour contaminated with seed	Toxic pyrrolizidine alkaloids in feed can kill livestock	Important weed reducing yields in cereal crops	Outcompetes annual plants, toxic to native mammals
*Ambrosia artemisiifolia*	Common ragweed	Highly allergenic pollen can induce allergic rhinitis	A cause of obstructive airway disease in horses	Important weed reducing yields in cereal crops	Competes with native plants and can alter biodiversity
*Vespula vulgaris*	Common wasp	Stings are painful and can lead to anaphylactic shock	Wasps attack honeybees and destroy hives	Wasps reduces bee pollination of crops	Compete with native insects and birds for insect prey
*Solenopsis invicta*	Red imported fire ant	Multiple painful stings often require medical attention	Nuisance pest of livestock, especially confined animals	Feeds on plants, and seeds and can girdle citrus trees	Reduces diversity of insects particularly other ant species
*Achatina fulica*	Giant African land snail	Vector of rat lungworm to humans	Host of several pathogens and parasites of livestock	Crop damage and vector of plant pathogens	Outcompetes native gastropods
*Branta canadensis*	Canada goose	Public health risk of fecal matter in waterbodies	Subject to avian influenza and could spread disease	Economic damage to crops on farmland	Aggressive and competitive toward native wildfowl
*Sturnus vulgaris*	European starling	Implicated in transmission of numerous human diseases	Transmit *Escherichia coli* O157:H7 from farm to farm	Costs millions of dollars in agricultural damage annually	Contributed to the decline of local native bird species
*Rattus norvegicus*	Norway rat	Host the plague bacterium *Yersinia pestis*	Spread livestock disease by contaminating feed with urine	Damage crops and stored grain	Lead to local extinction or range reduction of species
*Sus scrofa*	Feral pig	Carry trichinosis which is potentially fatal in humans	Reservoir host of foot-and-mouth disease	Extensive damage to agricultural crops	Rooting behavior disturbs soils
*Procyon lotor*	Raccoon	Host of rabies	Transmits canine distemper or raccoon roundworm	Significant cause of crop damage	Predation on a wide range of vertebrate prey

*Note:* Information for each species is drawn from the relevant datasheets of the CABI Compendium (CABI 2024).

Increasing awareness of biosecurity across multiple sectors should stimulate effective informed decision-making. Biosecurity risk assessments of alien species need to more explicitly consider multisectoral impacts, although such assessments are often undertaken with one sector firmly in mind (often plant health) rather than exploring the potential cascade of impacts over the longer term on the environment and public health (Roy et al. 2018). In contrast, public health practitioners have limited awareness of the role alien species might play in transmitting pathogens and parasites in their region or how such species can exacerbate symptoms of disease. This is in part due to a lack of large-scale data that report health outcomes of human populations over a long enough period prior to and then following an invasion. Building a robust system to assess this breadth of risks will be challenging but will likely be essential to the execution of One Health.

A fundamental feature of invasive alien species that affect multiple sectors is that often the breadth of their impacts is underestimated (figure 4). For example, the passionvine hopper (*Scolypopa australis*) was unintentionally introduced into New Zealand from Australia in 1880, and although it is thought of primarily as a pest of horticulture (especially kiwi fruit), it also feeds on the poisonous native tutu shrub (*Coriaria arborea*) concentrating the plant neurotoxins (tutin) in its honeydew, which is then collected by introduced honeybees, resulting in toxic honey and human poisoning (Chernyshev 2017). The introduction of the Nile perch (*Lates niloticus*) into Lake Victoria resulted in the establishment of a major fishery, as well as the extinction of several endemic fish species, but large-scale migrations of workers to the fishery in Uganda combined with patterns of sexual behavior among men and women involved in fishing, trading, and servicing the industry promoted the spread of HIV among the fishing communities, where its prevalence is three times the national average (Seeley et al. 2009). The multiple successive introductions of alien species can also progressively establish a web of interactions that encompass impacts across multiple sectors. European hares have become widely established in New Zealand following their introduction in 1851, and although their initial impacts were on vegetation, they became the primary wildlife host of the Asian longhorned tick following its import into the country in the late nineteenth century. Although initially causing only minor livestock losses, impacts became more significant following the introduction in 2011 of the protozoan parasite *Theileria orientalis* responsible for theileriosis in cattle for which the tick is the sole vector (Heath 2016).

**Figure 4.**
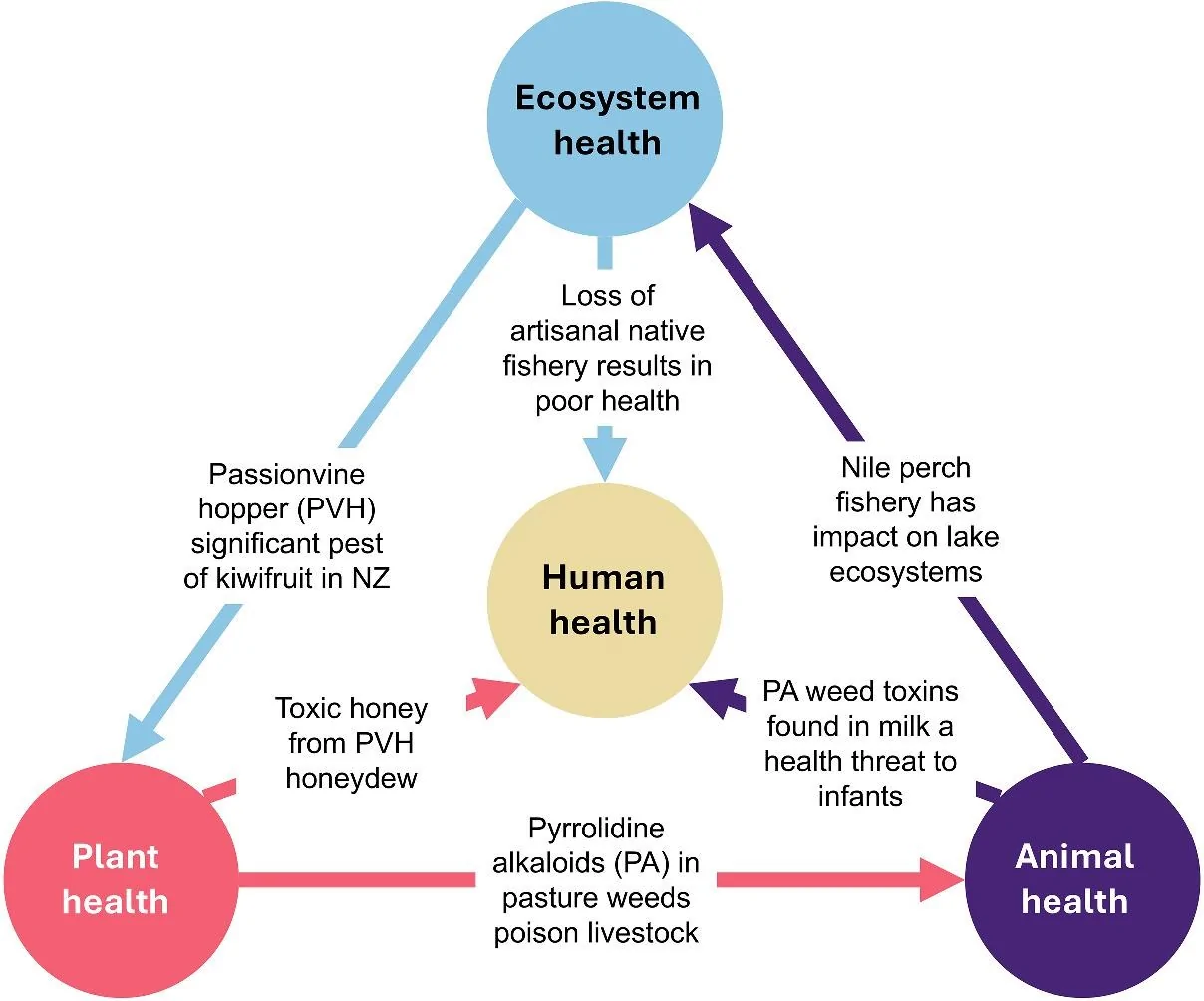
Illustration of how direct impacts of biological invasions on animal, plant, and ecosystem health can also have indirect and often unexpected impacts on human health. The color of the arrows refers to the corresponding sector (plant, animal, or ecosystem) where interventions to manage the invasive alien species would be required to prevent human health impacts. The examples include the direct effect of the passionvine hopper in New Zealand on kiwifruit health as a vector of plant pathogens but also its indirect effect on human health through the contamination of honey for human consumption with the neurotoxin tutin, the direct effect of the alkaloids in jimson weed affecting animal health when consumed by livestock in feed but also the human health impacts through contamination of milk for human consumption, and the direct effect of Nile perch on cichlid diversity in Lake Victoria and its indirect effect on human health through changes in social interaction in fishing communities.

These examples illustrate the complex outcomes that result from biological invasions initially viewed as affecting plant or ecosystem health also leading to unexpected human or animal health impacts. Acknowledgment of the multitude of potential interactions and complex feedback between human, animal, plant, and ecosystem health is not evident within the One Health literature but does sit at the core of One Biosecurity (Hulme 2021).

## Implementing One Biosecurity to support One Health

As we have outlined through the examples above, One Biosecurity is a fundamental component underpinning One Health because it bridges the human, animal, plant, and ecosystem health sectors presenting substantial opportunities for interdisciplinary knowledge generation. Nevertheless, to be effective there should be a clear implementation pathway to enable One Biosecurity to support One Health. Hulme (2021) described the potential for One Biosecurity to deliver dividends for both human health and the management of biological invasions at a global scale through a stronger regulatory instrument and the establishment of a multilateral biosecurity convention. However, the opportunities for One Biosecurity to help implement One Health at a national scale have not yet been explored. The biosecurity continuum (Gordh and McKirdy 2014) describes processes and interventions before the border (general surveillance and forecasting a threat prior to its arrival in a region), at the border (involving inspection and quarantine procedures), and after the border (early detection and rapid outbreak response and management). It presents a suitable framework for coordinated approaches across multiple sectors and scales that could deliver increased gains for One Health than at present. Seven areas are described that draw on examples from both biological invasions and public health to illustrate the utility of this framework that is inherent in the concept of One Biosecurity (figure 5)

**Figure 5.**
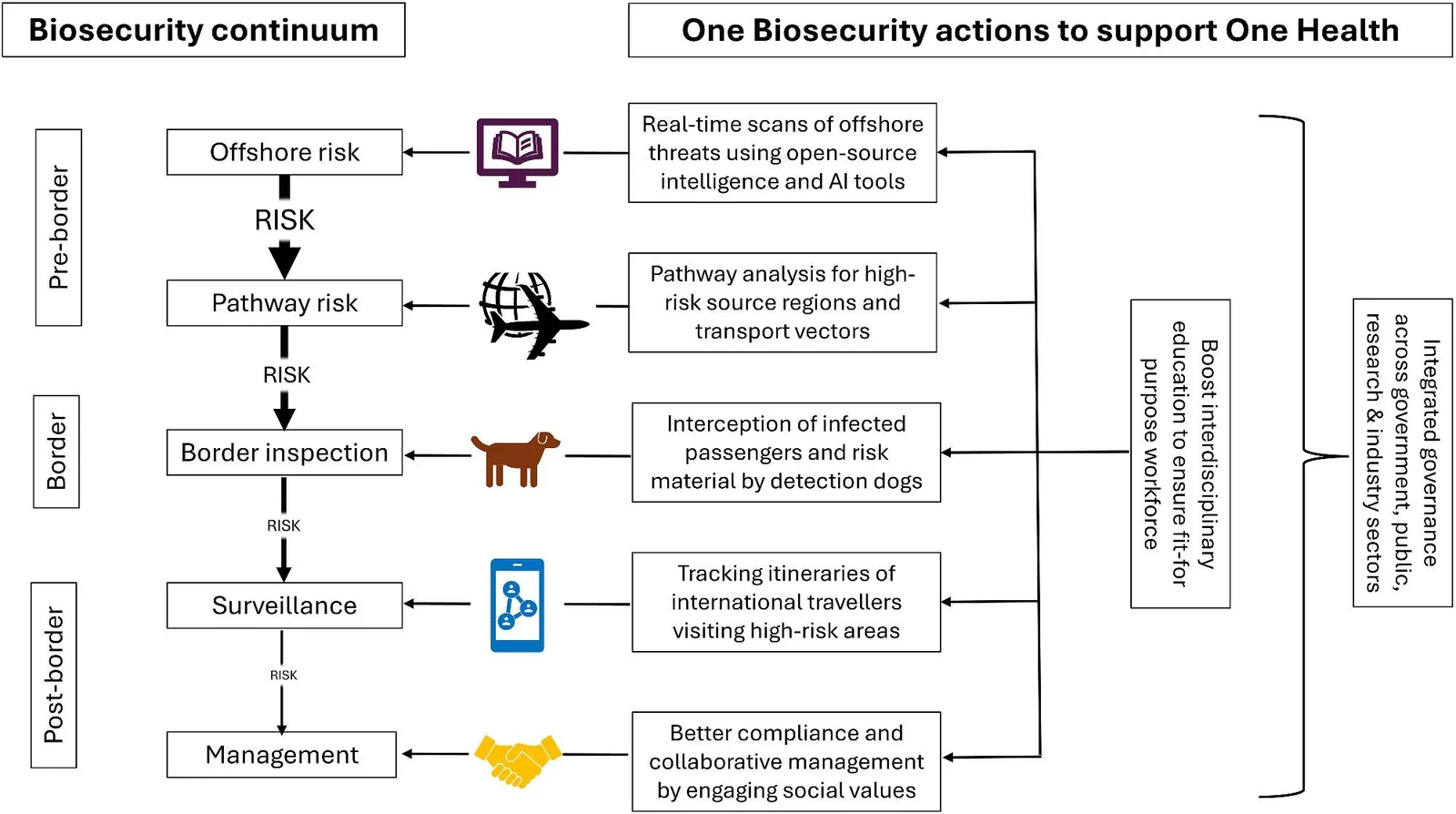
Schematic representation of activities undertaken along the biosecurity continuum that reduce the risk of an alien species incursion or a disease outbreak, highlighting seven specific One Biosecurity actions that would support One Health. These activities would involve approaches common to both public health and biological invasions before the border, at the border, and beyond the border and are meant to be illustrative rather than exhaustive. The essential message of One Biosecurity is that these activities need to be viewed as part of a whole package and therefore require integrated governance.

### Open-source intelligence to scan for emerging species threats across multiple sectors

Across all sectors, whether human, animal, plant, or ecosystem health a fundamental component of managing future risks is knowledge of emerging threats. The most effective means to address these threats is to prevent their arrival by knowing their likelihood of introduction (figure 5, pre-border offshore risk). If the likelihood that an alien species will be introduced is known, preventative measures can be implemented and the threat effectively addressed. Although there are a wide range of foresight methods available to forecast future threats (Hulme 2025), the most pressing need is for tools that can provide rapid (and frequently updated) real-time analysis of newly emerging pests, pathogens, and weeds. Increasingly open-source intelligence (OSINT) tools are being deployed to gather and analyze publicly available information (drawn from social media sites, news item, blogs, e-commerce platforms, etc.) to assess threats to public health (Bernard et al. 2018, MacIntyre et al. 2023) and risks from invasive alien species (Grossel et al. 2017, Tateosian et al. 2023). Despite the promise of automated systems scanning a wide range of digital information sources and feeding information into artificial intelligence algorithms that screen the data to present details of emerging risks many tools have become obsolete because of their cost and low success rate (Hulme 2025). Furthermore, even OSINT systems aiming to deliver similar global surveillance for emerging infectious diseases, such as HealthMap and the WHO Epidemic Intelligence from Open Sources, draw from different information sources and present conflicting estimates of risk (Ganser et al. 2022). A One Biosecurity approach could secure a longer-term future for such systems by consolidating OSINT tools across different sectors and data sources so that searches pick up not only emerging infectious diseases but also alien species. This is entirely feasible as illustrated by MEDISYS, a fully automatic surveillance platform retrieving news articles in order to detect emerging threats to public health in Europe that has recently been customized and expanded to cover plant and animal health (Mannino et al. 2021). By avoiding duplication and aiming for standardized information delivery, a cross-sector OSINT platform would facilitate the cross-referencing of different lines of information to provide a more complete picture of emerging risks such as where new records of multiple stings on the lower limbs of children (a public health notification) might point to the local establishment of the red imported fire ant (an invasive alien species of concern).

### Integrated analysis of shared introduction pathways

If knowledge of the emergence of a biosecurity threat is obtained sufficiently early then the threat may be contained; otherwise, there is a risk that the invasive alien species will spread to other regions. Managing these risks requires an understanding of how these biological threats might be introduced in a region, whether deliberately (perhaps as a form of bioterrorism) or accidently as a contaminant or stowaway on or in other products (figure 5, pre-border pathway risk). At a global scale, the spread of coronavirus (Ganser et al. 2022) and alien plant pests (Tatem 2009) have both been modeled in relation to the worldwide flows in the airline passenger network. Other vectors for the long-distance movement of organisms include migratory birds and the ballast water in ships, the former being associated with zoonotic diseases, whereas the latter is a pathway for coastal invasions. Although interest has been shown in modeling migratory pathways of birds as a potential route for the spread of avian influenza (Fourment et al. 2017), birds also vector alien species such as ectoparasites (Pandey et al. 2022) and commensal species (Costa et al. 2019) However, the opportunities to explore bird migration data to model the spread of organisms other than microbes remains underexplored. Similarly, although shipping routes have been the focus for modeling the global distribution of alien species in ballast water (Seebens et al. 2013), this is also a pathway for the introduction of human pathogens (Ruiz et al. 2000). Despite drawing from similar information sources, approaches to assess risks through introduction pathways have developed independently in the areas of human, animal, plant, and ecosystem health sectors. But at the core are a series of fundamentally similar questions that relate to the likelihood of the uptake of the taxon at the origin, its survival during transit, its chance of detection by public health officials or quarantine officers at the border and the prospect of establishment at the destination (Hulme 2009). A One Biosecurity approach would facilitate the sharing of species dispersal models (Gottwald et al. 2019, Thompson and Brooks-Pollock 2019) and encourage the wider availability of underlying data on different long-distance vectors, such as flight or shipping schedules to foster a global community working on these problems.

### Coordinated surveillance at the border for pests, pathogens, and weeds

Whether a commodity, a container or a passenger, biosecurity screening is often undertaken on arrival at a destination, usually at an administrative boundary or political border. Even with effective pre-border interventions, there is a need for border inspection, especially for cryptic organisms (figure 5, border inspection). Nucleic acid based environmental metabarcoding (eDNA and eRNA) provides an opportunity to identify potential biosecurity threats to human, animal, plant, and ecosystem health at the border (Hulme et al. 2023). Methods have been developed for cost-effective sampling of aircraft wastewater to assess the entry of human pathogens into a country from a known departure point (Bivins et al. 2024), as well as for sampling dust in shipping containers to detect plant pests (Trujillo-González et al. 2022). There therefore appears ample opportunity for eDNA tools to support One Health through a biosecurity surveillance context. However, such tools, although they are often effective, require investment in infrastructure and trained personnel for which resources may not be available in many countries.

A viable effective alternative to using eDNA is to employ detection dogs (Collins et al. 2022, Whitehead et al. 2024). Detection dogs detect biosecurity threats using the odor of volatile organic compounds emitted by risk material. They are increasingly employed to identify risk material being brought in by passengers at international borders (Moser et al. 2023), as well as to detect invasive alien insects (Hoffmann et al. 2022), weeds (Goodwin et al. 2010), and wildlife diseases (Golden et al. 2024) beyond the border. The opportunity to use detection dogs to screen for human disease among international travelers at the border also appears to be a viable, cost-effective screening option (Otto et al. 2021). Nevertheless, at the moment, those employing detection dogs to identify threats to human, animal, plant, and ecosystem health are not sharing their knowledge across sectors, and this limits the development of this area as a tool for One Health. A One Biosecurity approach would facilitate collaborative research to accelerate the effective use of detection dogs through internationally accredited training programs (for dogs and handlers), initiatives for improved dog breeding and welfare, and more effective operational methods that optimize the sensitivity and specificity of detection.

### Assessing risks of post-border introduction and spread of pests, pathogens, and weeds

The SARS-CoV-2 pandemic brought home the important role of human mobility in the spread of coronavirus and how certain individuals, locations, and activities could result in superspreader events (Loo et al. 2021). Understanding how an alien microbe, plant, or animal might disperse or be accidentally spread beyond the border is essential to plan any containment or rapid response initiatives (figure 5, post-border surveillance). Capturing the network topology of post-border movements by international travelers is a key element of biosecurity awareness whether it is addressing the risk of visitors bringing pests and pathogens into farms, weeds into national parks, or human pathogens into vulnerable communities. Irrespective of whether the risks are posed to human, animal, plant, or ecosystem health, to determine the likelihood of a biosecurity incursion it will be important to know the distribution of travel distances covered by individual travelers, the locations that attract the most visitors, the activities at a location that will best facilitate transmission, and how vulnerable locations are to a biosecurity threat. Tracking individuals raises many privacy concerns, and there is no overwhelming evidence to suggest that the intrusive contact tracing apps on mobile phones were especially effective at the height of the SARS-CoV-2 pandemic (Littlecott et al. 2023, Pozo-Martin et al. 2023). Rather than mobilize resources during an outbreak, there is a logic to capturing the broadscale trends in human mobility to prepare for future biosecurity threats. Such information can be captured passively by mining publicly available user-generated social media content or data generated automatically by mobile devices (Chen et al. 2024). In some countries, a stratified sample of departing international travelers is interviewed to gain insights into length of stay, sites visited, and activities undertaken, and these can be used to capture mobility patterns with a greater level of detail than social media posts (Hulme 2024a). Although such data may not be immediate enough to trace an ongoing outbreak, the information does provide a means to profile the characteristic of high-risk travelers (on the basis of age, nationality, etc.), as well as identify localities with high risk of transmission or vulnerability to impacts that should perhaps be the focus for surveillance. The common need for capturing human movements and modeling risks of transmission highlights the opportunities for a more joined up One Biosecurity approach.

### Social license for vaccination, pesticide application, and culling of pests

The tools used for the management of human and animal diseases, such as vaccination and antimicrobials, often differ from those applied to combat threats to plant and ecosystem health—for instance, pesticides, toxic baits, and culling. Despite these differences, commonalities exist in the deployment of such tools. For example, the strategies of implementing ring vaccination or establishing a cordon sanitaire to manage human infectious diseases have parallels in the management of invasive alien species where control efforts are implemented to establish a containment zone around an outbreak of an invasive alien species (Grice et al. 2020, Hulme et al. 2020). Irrespective of the tools and strategies used, an essential component of any eradication or management campaign is positive engagement with the public to build trust, foster acceptability of the campaign, and encourage participation (figure 5, post-border management). Recognizing the social and psychological impact of a disease or pest outbreak, and its subsequent management on affected stakeholders can help build supportive social values and cooperation that may reduce operational costs by encouraging greater compliance and even voluntary actions of citizens to protect shared values (Hulme et al. 2023). Furthermore, communities that have had a positive experience of a management campaign in one sector (e.g., successful eradication of a crop pest) may in the future be more willing to accept interventions targeting a different sector (such as an infectious disease in humans). Common issues that might underpin the public concerns with vaccines, pesticides, and gene technologies such as mistrust of government and scientists, misinformation, and uncertainty aversion need to be addressed long before any eradication campaign is set in motion. This may be best achieved by community engagement and awareness raising through a One Biosecurity approach across the human, animal, plant, and ecosystem sectors.

### Interdisciplinary biosecurity curricula for a skilled One Health workforce

Supporting the implementation of One Health across the biosecurity continuum described above will require a skilled workforce to respond to the increasing exposure of society and the environment to biological threats. Therefore, developing education and training programs that deliver high standards of professional excellence in biosecurity is an essential step in One Biosecurity (figure 5). Unfortunately, a more holistic understanding of biosecurity capacity building is challenged by strong sectorial identities associated with specific international standards and specialized research communities (Hulme 2020). Consequently, although a range of professional qualifications addressing biosecurity already exist, they are more in keeping with the biosafety of new technologies rather than the skillset needed to secure animal and public health or to protect biodiversity and the provision of ecosystem services (Moritz et al. 2020). Similarly, proposals for One Health curricula to support learning and professional development often ignore the role of biosecurity and biological invasions (Frankson et al. 2016, Vicente et al. 2021). Academic institutions and employers must recognize that biosecurity is a multidisciplinary field underpinning One Health and draws on a wide range of subjects including epidemiology, pathobiology, economics, social behavior, and invasion science that should be evident in the curricula employed in undergraduate and postgraduate training (Hulme 2024b). Rather than relying on a set of narrowly focused credentials, implementing a much broader multidisciplinary curriculum as a foundation for biosecurity professionals will be essential to strengthen the world's ability to prevent, detect, and respond to biosecurity threats worldwide. At the heart of such training programs should be the concept of One Biosecurity, which emphasizes the critical role biological invasions play both directly and indirectly in animal and human health and the range of skills needed for implementation along the biosecurity continuum. Governments and universities may be slow to shift their existing mindsets, but opportunities exist for partnerships involving the public, private, and academic sectors to be built around shared interests in biosecurity education, research, outreach, and implementation that would help support a biosecurity informed One Health curriculum (Hueston 2017).

### Adaptive and integrated governance of both biosecurity and One Health

Effective coordination, collaboration, and resourcing are essential to bring together the activities of multiple stakeholders across the biosecurity continuum to deliver One Health. The requirement for integrated governance has been identified as a key component to address both biological invasions (Roy et al. 2024) and One Health (Faijue et al. 2024). The shared need for the development of coherent national strategies, enhanced engagement with stakeholders (including indigenous and local communities), investment in research and technology, and the transparent collection and sharing of information highlight the potential synergies in delivering integrated governance for biosecurity and One Health (figure 5). Different government departments or ministries responsible for human health, agriculture or the environment often operate under separate legislation and, as a result, policy initiatives, investment in research and data gathering are rarely coordinated or shared. Such a situation leads to piecemeal initiatives, duplication of effort, wasted resources, and even perverse policy outcomes (McGeoch et al. 2023). International collaboration is critical in this regard, because some countries may have a much more developed institutional and legal framework for dealing with biosecurity, whereas others are just starting to consider the problem (Nunez and Pauchard 2010, Schwindt et al. 2024). Target 6 of the Montreal–Kunming Global Biodiversity Framework may be a good starting point for such enterprise as it requires much more ambitious national efforts to achieve the reduction of invasive alien species establishment and impacts on nature and society (CBD and IUCN 2024, Hulme et al. 2025).

The biosecurity continuum provides a framework for bringing the human, animal, plant, and ecosystem sectors together but also clearly identifies that governance of activities before the border, at the border, and after the border needs to be integrated. This requires an understanding of how to manage risks at multiple different scales from international (such as responding to international health regulations or trade agreements), to national (taking a whole of government approach to biosecurity), and then regional as well as local scales (where public engagement is essential). Consequently, biosecurity plays out across interconnected social, political, and environmental domains that are nested within each other and highly dynamic requiring an adaptive approach to governance that recognizes such dynamism and the need for holistic management (Rawluk et al. 2021). The multiple benefits of a One Biosecurity approach through adaptive and integrated governance could provide a stronger driver for governments and the international community to invest and work more closely across sectors to ensure prevention is a much stronger priorities across human, animal, plant, and ecosystem health.

## Conclusions

We present strong evidence as to why One Health should incorporate the interdisciplinary One Biosecurity approach. Claims that biosecurity is already integral to One Health are easily refuted by the multiple lines of evidence confirming its restricted application to farm hygiene, management of zoonoses and laboratory biosafety. In contrast, a One Biosecurity approach will not only ensure that One Health explicitly includes the role biological invasions play in human, animal, plant, and ecosystem health but also leverages existing biosecurity legislative and regulatory instruments at global and national scales to deliver greater public health benefits. One Biosecurity will also facilitate a clearer implementation plan across the biosecurity continuum. This includes the application of open science intelligence tools to forecast offshore risks, management of international pathways of people and commodities through pre-border inspections and surveillance of their subsequent post-border itineraries, and a more concerted effort to engage with social psychologists to help understand the steps necessary for improved compliance with biosecurity regulations. Although multilateral organizations such as the FAO, WOAH, UNEP, and WHO have established broad principles of working together to achieve One Health this often does not reflect the governance of public health and biosecurity at national scales where human, agricultural, and ecosystem health are usually the domain of separate ministries or departments. One Biosecurity provides a framework for closer working relationships across government by making clear the links between invasive alien species and the health of humans and livestock. Furthermore, by bringing together different sets of expertise (e.g., public health specialists, invasion biologists, social psychologists, economists) to address these issues, One Biosecurity gives a stronger interdisciplinary direction to One Health research. To maintain the growing global momentum to adopt One Health worldwide requires tangible evidence that the concept helps deliver better health outcomes and is not simply a rebranding of business-as-usual activities. One Biosecurity may be the step change needed to deliver on this promise.

## Author Biography

Philip E. Hulme (philip.hulme@lincoln.ac.nz) is distinguished professor of plant biosecurity and director of the Centre for One Biosecurity Research, Analysis, and Synthesis; he is also affiliated with the Department of Pest-Management and Conservation at Lincoln University, in Christchurch, Canterbury, New Zealand. Michele S. Dechoum is a professor in the Departamento de Ecologia e Zoologia and in the Programa de Pós-graduação em Ecologia of the Universidade Federal de Santa Catarina, in Santa Catarina, Brazil. Franz Essl is a professor in the Division of BioInvasions, Global Change, and Macroecology in the Department of Botany and Biodiversity Research at the University of Vienna, in Vienna, Austria. Angeliki F Martinou is affiliated with the Laboratory of Vector Ecology and Applied Entomology, Joint Services Health Unit Cyprus, in Akrotiri; with the Enalia Physis Environmental Research Centre, in Nicosia; and with the Climate and Atmosphere Research Centre, at the Cyprus Institute, in Aglantzia, Nicosia, Cyprus. Aníbal Pauchard is a professor in the Laboratorio de Invasiones Biológicas, in the Facultad de Ciencias Forestales at the Universidad de Concepción, in Concepción, and with the Institute of Ecology and Biodiversity, in Santiago, Chile. Petr Pyšek is affiliated with the Czech Academy of Sciences, Institute of Botany, Department of Invasion Ecology, in Průhonice, and with the Department of Ecology in the Faculty of Science at Charles University, in Prague, in the Czech Republic. Helen E. Roy is affiliated with the UK Centre for Ecology and Hydrology, in Wallingford, and with the Centre for Ecology and Conservation, at the University of Exeter, in Penryn, England, in the United Kingdom. Andy W Sheppard is the chief research scientist at CSIRO, Black Mountain Laboratories, in Canberra, Australia. Montserrat Vilà is a research professor at the Estación Biológica de Doñana and associate professor at the Departamento de Biología Vegetal y Ecología at the Universidad de Sevilla, in Sevilla, Spain. John R. U. Wilson is affiliated with the South African National Biodiversity Institute, at the Kirstenbosch Research Centre, in Cape Town, and is a core team member of the Centre for Invasion Biology, in the Department of Botany and Zoology at Stellenbosch University, in Stellenbosch, South Africa.
